# Beyond concealment: Translating placebo research into migraine care

**DOI:** 10.1111/head.70091

**Published:** 2026-04-03

**Authors:** Julian Kleine‐Borgmann, Katharina Schmidt, Ulrike Bingel

**Affiliations:** ^1^ Department of Neurology, Center for Translational Neuro‐ and Behavioral Sciences University Hospital Essen Essen Germany

**Keywords:** clinical, ethical, migraine disorders, pain perception, patient‐centered care, placebos

Migraine trials consistently reveal high placebo response rates, in some cases approaching—or even exceeding—the treatment effects observed with active comparators. Though such responses hamper the assay sensitivity in randomized clinical trials (RCTs), they also underscore the brain's capacity to modulate the experience of pain and other symptoms through expectancy and context. Yet, exploiting this potential in clinical practice has been constrained by the necessity of concealment in traditional placebo use—an ethical barrier that open‐label placebos (OLPs) are uniquely positioned to overcome. Experimental and clinical studies have shown that knowingly taking a placebo can still evoke clinically relevant responses.^1^ The act of pill‐taking within an empathic and credible clinical context may activate self‐regulatory and expectation‐based (neural) processes, enhancing symptom appraisal and control.

In our recent two‐center RCT, 120 patients with episodic or chronic migraine received either standard preventive care alone or standard care plus an OLP twice daily for 3 months.^2^ Participants were explicitly informed that the pills contained no active drug and that any communication and interaction with the study team was controlled for in both treatment arms. Whereas add‐on OLP treatment did not significantly reduce the number of migraine or headache days, patients reported improvements in quality of life, pain‐related disability, and overall well‐being. These findings suggest that OLPs can alleviate the subjective burden of migraine, even without altering attack frequency, which represents a relatively objective endpoint. However, an ongoing debate in headache care focuses on prioritizing patient‐centered outcomes that measure aspects of migraine burden beyond attack frequency. In this context, the preferential effects of OLPs on disability and quality of life serve as meaningful indicators of treatment efficacy.

The mechanisms underlying concealed placebos have been extensively studied, with well‐characterized neuro‐psycho‐biological pathways such as the activation of the descending opioidergic pain inhibitory network in placebo analgesia.^3^ In contrast, the mechanisms of OLP efficacy remain incompletely understood: Though short‐term OLP treatment appears to engage similar pathways, the dynamic processes supporting longer‐term treatment trajectories have not yet been examined.^4^ Individual traits and contextual factors, including optimism, catastrophizing, and fear of pain, may further moderate the interindividual response.^5^ Key questions for future research concern the durability of OLP effects and whether they can be maintained through brief reinforcement strategies, contextual cues, or other expectancy‐updating interventions. Interestingly, placebo acceptability may also vary across cultural contexts, an aspect that warrants consideration, particularly in the design and interpretation of global migraine trials.

Understanding the practical and cultural factors that shape clinician and patient acceptance of OLPs will be crucial for any future implementation in routine care. Despite their conceptual appeal, barriers may hinder the adoption of open‐label placebos in routine care, including time constraints, limited training in expectation management, and clinician skepticism, particularly in busy clinical settings. However, the expanding evidence base indicates that placebo and expectation mechanisms meaningfully contribute to treatment outcomes, especially in pain conditions, underscoring the importance of patient‐centered communication and shared decision‐making for future clinical guidelines and health care professional training. Interdisciplinary collaboration among neurologists, psychologists, and ethicists will be essential to translate OLP research into clinical guidelines. Realizing the potential of OLPs in routine care will rely on participatory, patient‐centered approaches that foster openness, shared decision‐making, and trust. Qualitative research suggests that when these elements are insufficiently addressed, patients may perceive OLPs as dismissive or as undermining the seriousness of their condition; however, when presented transparently and empathetically, most patients view OLPs positively and express openness toward their use.^6^, ^7^

OLPs offer several advantages in medical care, including excellent safety with minimal side effects, full transparency in line with ethical standards, and high flexibility to complement both pharmacological and behavioral prevention strategies. They have the potential to serve as an additional option for patients who prefer to limit medication use, have experienced side effects, or seek a more holistic and participatory form of care. They may also foster greater engagement and empowerment in their treatment, supporting adherence to established therapies through positive expectations and a stronger sense of personal agency. Importantly, OLPs should not replace established preventive regimens but may enhance patient‐centered outcomes—a growing priority in migraine management.

Moreover, a promising future use of OLPs is placebo‐controlled dose reduction,^8^ where the dose of an active medication is gradually decreased. An OLP is used simultaneously to help preserve clinical benefits. This method is based on classical conditioning principles and has proven effective in reducing opioid use after spine surgery.^9^ Potential benefits include fewer side effects, better adherence, maintained efficacy at lower doses, and lower treatment costs.

Importantly, the implications of this research extend far beyond placebo pills. Placebo effects and their negative counterpart—nocebo effects—permeate all therapeutic encounters and substantially modulate the efficacy and tolerability of pain treatments and beyond. Expectations as their key determinant of symptom perception and treatment response are not bound to placebo pills. Ethically harnessing placebo effects requires optimizing the therapeutic setting by examining current treatment expectations, previous treatment experiences—whether positive or negative—clearly communicating expected treatment effects and benefits, and carefully balancing information about potential adverse events with treatment advantages. It is important to emphasize that systematically utilizing these effects does not in any way undermine the importance of headache medicine or the clinician's role. Instead, it underscores a specific clinical skill that enhances treatment delivery and promises to improve the effectiveness and tolerability of existing interventions with implications far beyond OLPs themselves. Patients' expectations can—and should—be deliberately targeted as part of any therapeutic approach.^10^ Ultimately, well‐structured, accessible, and patient‐centered communication remains one of the most powerful tools in medicine, with or without a placebo.

## AUTHOR CONTRIBUTIONS

**Julian Kleine‐Borgmann:** Conceptualization; writing – original draft; funding acquisition. **Katharina Schmidt:** Writing – review and editing; funding acquisition. **Ulrike Bingel:** Resources; writing – review and editing; supervision; funding acquisition.

## CONFLICT OF INTEREST STATEMENT

**Julian Kleine‐Borgmann** and **Katharina Schmidt** have no conflicts of interest to declare. **Ulrike Bingel** reported receiving grants from the German Research Foundation during the conduct of the study and personal fees from Grünenthal, Apurano, Roche, and Biogen outside the submitted work.
